# Giant Multinucleated Cells Are Associated with Mastocytic Inflammatory Signature Equine Asthma

**DOI:** 10.3390/ani12091070

**Published:** 2022-04-20

**Authors:** Ilaria Basano, Alessandra Romolo, Giulia Iamone, Giulia Memoli, Barbara Riccio, Jean-Pierre Lavoie, Barbara Miniscalco, Michela Bullone

**Affiliations:** 1 Department of Veterinary Sciences, University of Turin, 10095 Grugliasco, Italy; ilaria.basano@unito.it (I.B.); alessandra.romolo@edu.unito.it (A.R.); giulia.iamone@unito.it (G.I.); giulia.memoli@unito.it (G.M.); barbara.riccio@unito.it (B.R.); barbara.miniscalco@unito.it (B.M.); 2 Department of Clinical Sciences, Faculty of Veterinary Medicine, Université de Montréal, Saint-Hyacinthe, QC J2S 2M2, Canada; jean-pierre.lavoie@umontreal.ca

**Keywords:** equine asthma, macrophage fusion, giant multinucleated cells, mast cells, bronchoalveolar lavage, cytology

## Abstract

**Simple Summary:**

Lung inflammation is commonly assessed in asthmatic horses by bronchoalveolar lavage fluid (BALF) cytology. Among the cell types commonly found in equine BALF samples, macrophages are the most abundant. The clinical significance of the abnormal cytological appearance of macrophages in samples from diseased horses is largely disregarded. The present work focuses on cytological alterations observed in macrophages during a chronic inflammatory disease such as equine asthma. Our data, although limited in number, support macrophage fusion (resulting in multinucleated giant cells) as a mechanism of interest in the classification of equine asthma phenotypes, as it was significantly associated with the inflammatory signature and chronicity of the disease.

**Abstract:**

Equine asthma is currently diagnosed by the presence of increased neutrophil (>5%), mast cell (>2%), and/or eosinophil (>1%) differential cell count. Macrophages are normal resident cells within the alveoli. Their presence in BALF is considered normal, but the clinical implication of the presence of activated or fused macrophages (giant multinucleated cells, GMC) is currently overlooked. We aimed to assess the prevalence, cytological determinants, and clinical significance of increased GMC counts in BALF of 34 asthmatic horses compared to 10 controls. Counts were performed on 15 randomly selected high magnification fields per cytospin slide (40×), and expressed as GMC:single macrophage (GMC:M) ratio. Regression models were used for statistical analysis. GMC was frequently observed in both asthmatic and control horses, with an increased prevalence of equine asthma (*p* = 0.01). GMC:M ratio was significantly higher in severe vs. mild to moderate equine asthmatic and control horses. In asthmatic horses, an increased GMC:M ratio was significantly associated with BALF mastocytosis (*p* = 0.01), once adjusting for age and the presence and severity of clinical signs of the horses. Tachypnea was the only clinical sign that tended to be positively associated with GMC:M ratio after adjustment (*p* = 0.08). In conclusion, our data suggest that a relationship might exist between molecular mechanisms regulating GMC formation and mast cell recruitment in the equine lung. The same mechanisms could lead to tachypnea even in the absence of respiratory effort at rest. We suggest including GMC count in the basic cytological assessment of BALF samples to gain more insights into their role in equine asthma.

## 1. Introduction

Equine asthma is a highly prevalent disease in horses of all ages, characterized by chronic lower airway non-septic inflammation [1]. Equine asthma can manifest as different disease entities or phenotypes, based on severity and recurrence of clinical signs. The severe asthma phenotype (severe equine asthma, SEA) describes adult horses presenting with recurrent and reversible bouts of bronchospasm and respiratory effort at rest, often associated with cough, mucus nasal discharge, tachypnoea, and exercise intolerance. Severe asthma is typically associated with bronchoalveolar lavage fluid (BALF) neutrophilia >25% [2]. Sporadically, concomitant mastocytosis is also reported [3]. Mild to moderate equine asthma (MEA) describes a much broader clinical entity with no age predilection and clinical signs lasting for more than 3 weeks. Clinical signs span from poor performance in racehorses to more obvious cough, nasal discharge, or dyspnoea during or after exercise in show horses. The MEA phenotype is currently classified based on the inflammatory signature observed in BALF as neutrophilic (if neutrophils are >5% at differential cell counts), mastocytic (>2%), eosinophilic (≥1%), or mixed [1]. There is substantial agreement on the fact that both genetics and environmental factors play a role in equine asthma pathogenesis and pathophysiology [4,5,6]. A large amount of evidence supports the primary role of straw and hay dust (and/or pollen) exposure as triggers for SEA exacerbations [7,8,9,10,11,12,13,14]. On the other hand, information on the etiology of MEA cases is scarce. Whether an initial trigger is needed for disease development is unknown in both conditions.

Cytological assessment of BALF is considered the gold standard technique for the diagnosis of equine asthma, together with relevant and compatible clinical signs [1]. The presence of qualitative and/or quantitative alterations of cytological entities other than neutrophils, mastocytes, and eosinophils is often disregarded in equine cytopathology. Nevertheless, it deserves attention as it might have clinical implications. Macrophages are normal resident cells within the lung, particularly within the alveoli. Their presence in equine BALF is physiological and their quantity has been shown to remain unchanged or decrease with aging [15,16]. It must be noticed that available data on age-associated changes in equine BALF differential cell counts are limited, and often restricted to secondary outcomes in relevant studies. Macrophages can exhibit various phenotypical features of activation, phagocytosis, and fusion, whose significance is currently overlooked. Monocyte/macrophage fusion results in giant multinucleated cells (GMC) and is considered a marker of chronic inflammation. In tissues, they are assumed to represent a specialization for improved phagocytosis (i.e., during a foreign body response) [17]. Molecular pathways leading to GMC formation are many and represent an emerging research field [17]. It is overall recognized that interleukin (IL)-4, a Th-2 cytokine whose role has been highlighted also in SEA [6,18,19], is likely to play a major role in this process [20]. Of note, IL-4 is implicated in mast cell recruitment and activation as well [21].

With the current work, we aimed at investigating the prevalence of GMCs in the BALF of asthmatic horses, as well as their association with relevant clinical or cytological features of equine asthma. Our hypothesis was that GMCs are increased in horses with equine asthma vs. controls, and their number is associated with disease chronicity (namely, with a diagnosis of SEA) and BALF mastocytosis.

## 2. Materials and Methods

### 2.1. Study Design

Cases referred to the Veterinary Teaching Hospital of the University of Turin with a diagnosis of equine asthma as reported in the electronic medical record file were searched retrospectively on April 2021. Additionally, newly referred cases diagnosed with equine asthma were prospectively included starting from April 2021. Cases were included in the study if the information on history, first examination, bronchoalveolar lavage procedure, and results were reported, and if BAL cytology slides were still available for re-assessment. Records were reviewed and cases with incomplete information or with uncertain diagnoses were excluded. Additionally, horses with concomitant positive bacterial culture from trans-tracheal wash samples and horses that had corticosteroids or antimicrobial treatments in the 2-week period preceding BALF sampling were excluded. Asthma cases (SEA and MEA) were defined following the current revised ACVIM Consensus Statement [1]. Briefly, SEA cases were defined based on relevant history and clinical signs; mild to moderate equine asthma cases were defined as those with relevant clinical signs and BALF neutrophil >5%, eosinophils >1%, and/or mast cells >2%. Mastocytic mild to moderate asthma (M-MEA) was defined based on the finding of >2% mast cells in BALF, in the absence of any other cytological abnormality [1], in horses with relevant respiratory signs. The following data were obtained from medical records concerning the horses studied: age, sex, relevant clinical signs (in particular: cough, nasal discharge, dyspnea, and poor performance), and time of the year when the exam was performed (month). Cytospins of all cases were reviewed for a blind reassessment of differential cell counts by the same operator and GMC quantification. The study was approved by the Local Animal Ethical Committee of the Department of Veterinary Medicine (Prot. N. 711, 17 March 2021), University of Turin. Written informed consent was obtained from the owners to use clinical data for research purposes.

Further asthmatic and control horses were retrospectively enrolled among equine patients referred to the Veterinary Teaching Hospital of the University of Montreal, Canada. Data and BAL cytospins slides of this second population of horses were retrieved by the Equine Respiratory Tissue Bank (https://btre.ca/, accessed on 1 November 2021). Horses were randomly selected among those available based on their diagnosis (5 SEA, 5 MEA, 10 controls defined as healthy or with musculoskeletal disease).

### 2.2. Bronchoalveolar Lavage Procedure and Processing

The BAL procedure was performed as described by Hoffman [22] on standing sedated horses restrained in a stock. Briefly, two boluses of 250 mL warm sterile saline solution were instilled in the distal airways and lung parenchyma by means of a Bivona catheter and gently withdrawn. A 5 mL-aliquot obtained after gentle resuspension of the entire solution withdrawn was immediately submitted for cytology in an EDTA tube. Cytospin slides (n.2) were prepared using 200 µL of unfiltered fresh samples centrifugated at 500 RPM for 5 min at room temperature. Slides were air-dried and stained with May-Grunwald-Giemsa using an automated slide stainer (Mirastainer^TM^ II System, EMD Chemicals Inc., Darmstadt, Germany).

The methods used in Turin and Montreal [2] for BALF fluid withdrawal and analysis differed only for the use of the Bivona catheter (3 m in length, 11 mm outer diameter) or Olympus SIF-Q140 videoendoscope (working length 250 cm, 10.5 mm outer diameter) to reach the sampling site.

### 2.3. Cytology

Cytospins slides were available for all horses studied. Slides were assessed by the same operator (AR), blinded to the animal ID, who performed: (1) differential cell counts for neutrophils, lymphocytes, macrophages, mast cells, and eosinophils; and (2) GMC counts. We defined GMC as cells with 2 or more nuclei and with evidence of unique cytoplasm.

(1) Differential cell counts were performed at 40× magnification on a minimum of 500 cells and 5 high power fields (HPF) [23]. Epithelial cells were excluded, when present and GMC were counted as macrophages (i.e., a GMC with two nuclei accounted for two macrophages).
(2) Counts of GMC were performed at 40× magnification over 15 HPF and expressed as GMC:single macrophages (GMC:M) ratio. They were obtained from:

$$GMC:M_{horse}=\frac{\sum_{i=1}^{n=15}{\left(GMC\right)}_{i}}{\sum_{i=1}^{n=15}{\left(M\right)}_{i}}$$

where *i* represents HPF and *n* is the minimum number of HPF per slide that must be assessed for a reliable estimate. To determine *n*, the absolute number of GMC and single macrophages were initially counted in all non-empty HPF of a subset of 10 samples (from 5 MEA and 5 SEA horses).

### 2.4. Statistical Analysis

Data were analyzed with GraphPad Prism version 8.0.0 for Windows (GraphPad Software, San Diego, CA, USA) and with STATA 15 software (StataCorp 2017, Stata Statistical Software: Release 15, College Station, TX, USA; StataCorp LLC.). Data distribution was assessed with the Saphiro Wilk normality test before and after the log transformation of the data. As log transformation did not yield significant advantages in data distribution, raw data were used for analysis. Groups’ mean values were compared using the ANOVA test. Association between GMC:M ratio and other clinical or cytological/inflammatory parameters were assessed with linear or logistic regression models, depending on the nature of the independent variable (continuous or binomial, respectively). Data were adjusted for age (years) and for the presence and severity of clinical signs (for each clinical sign observed among cough, nasal discharge, tachypnea, increased respiratory effort at rest, this variable was increased by 1 point; the final variable could range from 0 to 4). Normalized cumulative average charts were used to determine the minimum number of HPF needed to accurately estimate the GMC:M ratio and its difference among the groups studied, under the assumption that an average 15% error is considered normal in manual cell counting. Cumulative average charts were normalized with respect to the grand average of the sample (equal to 1).

## 3. Results

### 3.1. Horses

A total of 42 horses were included in the study. Their clinical details are reported in Table 1.

Briefly, there were 32 asthmatic and 10 control horses. There were 18 Warmblood horses, 7 Standardbred horses (none of them actively racing, 6 used for leisure activities, and 1 stallion used only for breeding), 1 draft horse, 5 ponies, 2 Friesian horses, and 9 Quarter Horse or related breeds. The Turin cohort was composed mainly of horses with MEA (n = 14). Horses with MEA with no previous treatments presented mostly with mastocytic inflammation at BALF (n = 7), with a small percentage having pure neutrophilic (n = 2) or mixed neutrophilic and mastocytic (n = 5) inflammation. The Montreal cohort was composed of 10 control horses (of which, 5 were healthy and 5 with concomitant orthopedic problems), 5 MEA (3 neutrophilic, 1 mastocytic, and 1 paucigranulocytic), and 5 severe asthmatic horses (Table 2). Five out of ten control horses presented mild increases in inflammatory cell counts at BALF (Table 2). The paucigranulocytic IAD case was a 10-year-old female Warmblood used for dressage, diagnosed with MEA in Montreal. Medical records reported differential cell counts as: 5% neutrophils, 1% mast cells, 0% eosinophils, 41% macrophages and 54% lymphocytes. This mare was referred for chronic cough at the beginning of exercise and at rest in box, and prolonged recovery after exercise. The owner reported no exercise intolerance, nor was noticed the presence of respiratory effort at rest during a clinical examination. The horse had been showing these signs for 2–3 years before referral, and the owner reports clinical improvements while feeding wet hay and following bronchodilator and anti-inflammatory therapies in the past. Horses with MEA were presented for: cough (8/19), increased respiratory effort during or after exercise (8/19), nasal discharge (5/19), tachypnea (5/19), and poor performance (3/19). Two horses diagnosed with MEA from the Turin cohort (2 M-MEA and 1 mixed) and 1 horse from the Montreal cohort did not present any relevant clinical signs at the time of examination. They were however included due to the relevant history and clinical complaint. 

### 3.2. Giant Multinucleated Cell Counts

#### 3.2.1. Counting Method

**Figure 1:**
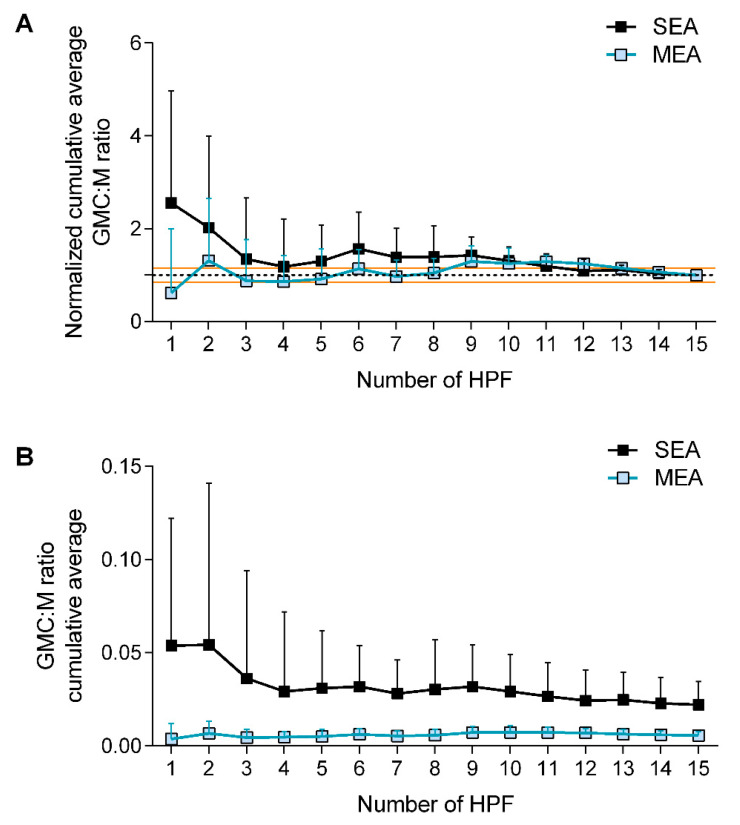
Validation of the methodology proposed for GMC counting in BALF cytological slides. (**A**) Normalized cumulative average curve shows the percentage variation of each cumulative measure against the total average (constrained to 1 and identified with the dotted line). Red lines identify the limits of the acceptable 15% error above or below the mean. (**B**) Cumulative average of GMC:M ratio over 15 measurements performed on 15 high power fields (HPF). SEA: severe equine asthma. MEA: mild to moderate equine asthma.

The normalized cumulative average chart of GMC:M values shows that starting from 13 HPF, the average error falls below 15%, suggesting that the assessment of 15 HPF per sample provides precise estimates (Figure 1A). Of note, initial variability was higher for SEA vs. MEA samples, possibly because GMC are unevenly distributed across the microscope slide or because of a subjective bias towards reading first the fields with the highest number of GMC. Further tests specifically assessing cell distribution on the slide and comparing subjective vs. computer-based randomized reads on the same slides might ascertain these points.

The counting method employed allowed the detection of significant differences between SEA and MEA horses using a subset of randomly selected 10 horses (5 per group). Data suggest that a reliable estimate of the difference between these two groups can be achieved by counting 5 HPF (Figure 1B). For the aim of the present study, all GMC:M counts were performed on a total of 15 HPF per slide, to increase the accuracy of the measurement.

#### 3.2.2. Disease Effect and External Validity of the Study

**Figure 2:**
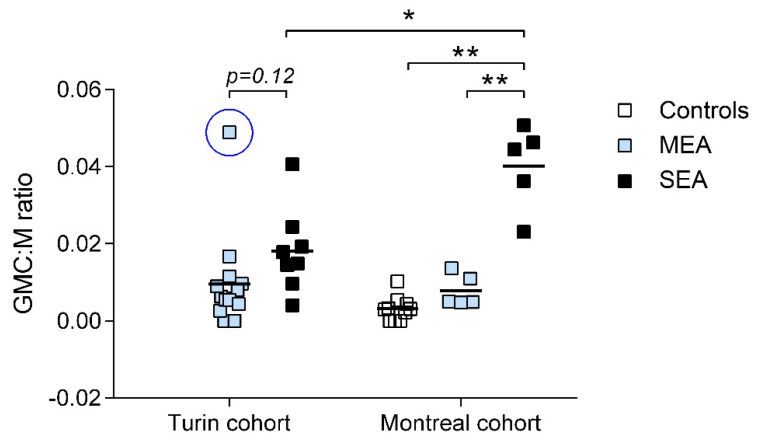
Effect of disease and cohort on GMC:M ratio (N = 42). *: significantly different (*p* < 0.001); **: significantly different (*p* < 0.0001). SEA: severe equine asthma. MEA: mild to moderate equine asthma. The blue circle identifies the outlier, whose clinical description is provided within the text.

Giant multinucleated cells were observed in 7/10 (70%) control horses, 17/19 (89%) mild to moderate, and 13/13 (100%) severe asthmatic horses. Overall, a progressive increase in GMC:M ratio was noticed in MEA and SEA horses compared to controls. Considering both cohorts in a two-way ANOVA model, a significant effect of disease (*p* < 0.0001) and of the cohort (*p* = 0.02) were observed on GMC:M ratio (Figure 2), as well as a significant interaction among these covariates (*p* = 0.004). Post-tests revealed significantly higher values of GMC:M ratio in SEA and MEA compared to control horses (*p* < 0.0001 for both). When the two cohorts were assessed separately, GMC:M ratio was higher in SEA vs. MEA only in the Montreal cohort, due to the presence of an outlier in the Turin cohort. This outlier was a 9-year-old female horse with mixed BALF inflammation (6% neutrophil, 3% mast cell count in BALF) referred for chronic mucous nasal discharge and tachypnea at rest. Severe equine asthma in this horse was ruled out based on the absence of response to bronchodilators and no response to a 10-day course of oral dexamethasone treatment (in the absence, however, of environmental improvements). The mare had normal lung radiography and ultrasound, tested negative for EHV5 on both trans-tracheal wash (TTW) and BALF samples, and TTW yielded no bacterial growth. Removal of this outlier from the analysis revealed a significantly higher GMC:M ratio in SEA vs. MEA also in the Turin cohort (*p* < 0.01). Based on the available data, we cannot exclude that the mare had SEA, but the lack of response to corticosteroid does not support this hypothesis. It is possible that a longer treatment time could have shown an effect. A higher GMC:M ratio was also noticed in SEA horses from Montreal vs. SEA horses from Turin, and this was not associated with a different clinical presentation, based on the information available in medical records. No difference was observed in the GMC:M counts between healthy controls and controls with orthopedic problems (data not shown).

Based on these results, horses from both cohorts were included in subsequent analysis to increase the power of the study. 

### 3.3. Giant Multinucleated Cell Counts Relationship with Clinical Parameters and Inflammatory Signatures

GMC:M ratio and the percentage of macrophages in BALF were both significantly associated with the age of the horses. Specifically, GMC:M ratio increased while the percentage of macrophages decreased with aging (*p* = 0.01 and *p* = 0.001, respectively). The association between GMC:M and macrophage percentage in BAL also showed a significant trend towards an inverse association (*p* = 0.05). Once adjusted for clinical signs, however, the association of GMC:M with both age and macrophages in BALF became not significant (*p* = 0.85 and *p* = 0.34, respectively). Contrarily, the adjusted approach revealed a significant decrease in the percentage of alveolar macrophages with aging (β coefficient = −1.06; 95% CI −2.00, −0.11; *p* = 0.03).

GMC:M ratio was not associated with any cytological variable in an unadjusted univariate analysis restricted to asthmatic subjects. Adjusting for the presence and severity of clinical signs and for age revealed a significant association between GMC:M ratio and the percentage of mast cells in BALF (Table 3). Given the presence of an outlier in the Turin cohort with a very high GMC:M ratio (0.0489) and 3% mast cells in BALF, analyses were repeated in the absence of this animal. Exclusion of the outlier confirmed the observed results concerning the association between GMC:M ratio and BALF mastocytosis (β coefficient = −0.0034826; 95% CI −0.0053072, −0.0016579; *p* = 0.001), and revealed a previously undetected significant association between GMC:M ratio and BALF neutrophilia (β coefficient = 0.0004044; 95% CI 0.0001487, 0.0006601; *p* = 0.004).

**Figure 3:**
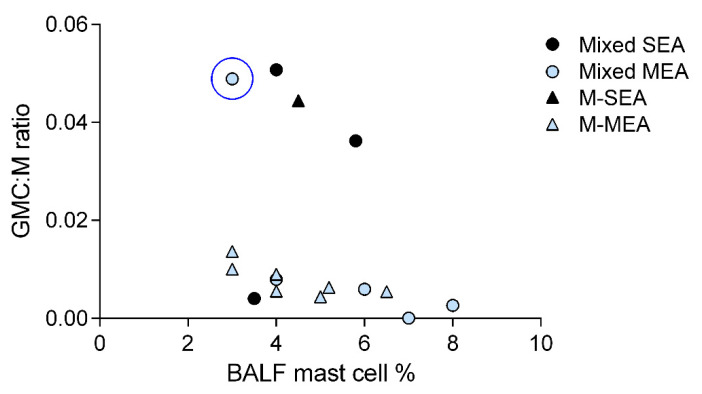
Relationship between GMC:M ratio and BALF mastocytosis in equine asthma (N = 17). Two clusters can be distinguished based on GMC:M ratio. In both, the relationship between GMC:M ratio and BALF mastocytosis is inverse. The blue circle identifies the outlier already identified in Figure 2, with a 1-year-history of tachypnea and mucous nasal discharge. Circles represent horses with mixed neutrophilic-mastocytic inflammation. Triangles represent horses with pure mastocytic inflammation. Black identifies SEA. Light blue identifies horses with MEA. Horses with no clinical signs at examination (n = 3) are not depicted to better represent the statistical model employed.

A graphical representation of the relationship between GMC:M and BALF mastocytosis is provided in Figure 3. This graph suggests the existence of two distinct cytological clusters that follow the same pattern (inverse relationship), possibly related to disease chronicity. Concerning the clinical signs presented by the horses studied, those significantly associated with the presence and quantity of GMC (expressed as GMC:M ratio) were found to be the presence of respiratory effort at rest (*p* = 0.01) and tachypnea (*p* = 0.007). Adjusting for the presence and severity of clinical signs completely abolished the significant association with an increased respiratory effort at rest, while a tendency to significance was still appreciable for tachypnea (*p* = 0.08).

## 4. Discussion

Increased attention has been paid to the role of alveolar macrophages in equine asthma in recent years, as they appear to behave as crucial regulators of both acute inflammatory response and its subsequent resolution [24]. Alveolar macrophages are normal inhabitants of the lower airways, and their fusion produces GMC [17]. Giant multinucleated cells are commonly observed in BALF samples from asthmatic horses, thus often disregarded. Like macrophages, different phenotypes of GMC are recognized in health and disease, some of which can be distinguished based on morphological features while others do not [17,25,26]. A deeper understanding of the role and activity of GMC in equine respiratory health and diseases might provide additional information in clinical settings. It might also help in the comprehension of physiopathological mechanisms driving disease development and maintenance, with potential implications for therapeutic advances. Our work provides the first insight into this complex panorama, suggesting that the presence of GMC in increased quantities is a feature of equine asthma, especially in its severe form. Moreover, the significant association with mastocytes suggests the interleukin (IL)-4 signaling pathway is involved, in accordance with previous studies identifying a central role of this cytokine in equine asthma [18,19,27].

The prevalence of horses with GMC in their BALF was high in our study, reaching 70% in control horses and almost 100% in asthmatic horses. This was unexpected but easily explained by the environment horses are exposed to and to the criteria we used to define GMC. Horses are naturally exposed to dusts of biological, vegetal, or mineral origin. It is reasonable to hypothesize that a dust-rich environment stimulates GMC formation in the lungs even in the absence of pulmonary diseases. Our definition of GMC as cells with two or more nuclei of monocyte origin was also loose. Data in human beings highlights that GMC with a low number of nuclei (>three) are highly prevalent in respiratory samples from healthy people and patients with chronic lung diseases. Contrarily, GMC with >10 nuclei were observed in only 10% of human pathological BALF samples, most often associated with granulomatous diseases (sarcoidosis, pneumoconiosis, interstitial lung diseases) or chronic non-interstitial lung diseases [25]. Equine asthma is not a granulomatous disease. However, cases characterized by miliary granulomatous lesions of unknown origin have been reported [28], which would be consistent with the finding of GMC in BALF. Idiopathic asthmatic granulomatosis has been described also in human beings [29,30]. Further work on alveolar GMC in healthy and asthmatic horses might be of value for establishing, respectively, normal values and expected ranges in this common disease. This approach could facilitate the identification of less common granulomatous or GMC-associated pulmonary conditions in horses.

Control horses included in our study did present cytological abnormalities. This is in line with previous observations in a population of actively performing show horses [16], and could be linked with the stabling environment [7,31]. We acknowledge that control animals were available from only one site in our study, and this is a limitation of our current approach. However, data from MEA and SEA horses from both sites are concordant, and there is no reason to expect that the lack of control horses from one site would have introduced a bias in our data. A further aspect that might have helped in understanding the causes of GMC formation in our population is the availability of environmental particle loads by air sampling. This is a piece of information that can be obtained quite easily, although it remains unspecific. A thorough characterization of the exogenous particles within alveolar macrophages and GMC would provide relevant information concerning the origin of GMC formation in our horses.

Macrophage fusion and GMC formation require cell priming and expression of adhesion molecules called fusogens [17]. Depending on the stimulus and mechanisms underlying macrophage fusion, different GMC phenotypes are currently recognized. Broadly, GMC are classified as classically activated and alternatively activated. Classically activated GMC are those typical of acute infective processes (typically mycobacterial ones), its development is driven by interferon (IFN)-γ, lipopolysaccharide (LPS), and tumor necrosis factor (TNF)-α. The major fusogen of this phenotype is the dendrocyte-expressed seven-transmembrane protein (DC-STAMP). Alternatively activated GMC are a wider family typical of sarcoidosis, foreign body, parasite reactions, and chronic tuberculosis. Alternatively activated GMC are mainly linked with a Th-2 type immune response (IL-4, IL-13 rich environment), but sub-phenotypes responsive to IL-10 or IL-6 are also recognized. They typically express fusogens as E-caderhin or a mannitol receptor (CD206) [17]. Recent work has shown that macrophage polarization occurs in equine alveolar macrophages following the same patterns described in human beings, with CD206 expression being significantly enhanced by IL-4 stimulation [27]. The same work, however, failed to show a significant increase in CD206 expression in asthmatic horses, either in remission or exacerbation of the disease, compared to control horses. Another study reports that CD206 expression assessed by flow cytometry was increased in alveolar macrophages of asthmatic horses during disease exacerbation vs. remission of the disease, although values of asthmatic horses were not different from controls [32]. Whether GMC formation was assessed and accounted for, was not specified in both papers. The results observed are in line with previous studies showing activation of different immune (T cell) responses in equine asthma cases [33], and highlight the need to find clinically applicable markers of such equine asthma endotypes that can be used together with BALF cell counts.

Quantification and interpretation of BALF cell counts, per se, remains challenging. BALF cell counts are commonly expressed in percentage, as the absolute number is dependent on many uncontrollable factors. This is especially relevant in equine medicine, in which the BAL technique is only partly standardized and data exists in support of significant effects on BALF cell counts induced by the volume of fluid instilled or by the counting method used [22,23,32,34]. The same is true for GMC quantification, for which only relative measures are available or commonly used [35]. In this perspective, an increase in GMC:M ratio as the one we observed in SEA could be driven by an increased fusion of macrophages or by a decreased number of alveolar macrophages. In turn, decreased macrophage count might have been linked with increases in other cell types, given their expression in relative units. The measuring unit we used did not permit the assessment of a parallel increase in both singular alveolar macrophages and GMC. GMC:M ratio was not associated with BALF macrophage percent count in our population, while significant associations were observed with neutrophils and mastocytes. Among several cytokines involved in neutrophil or mast cell recruitment, IL-4 is of particular interest as it has been shown to play a role in SEA-associated neutrophilia [19], it induces mast cell proliferation and survival through several pathways [36], and it is synthesized by mast cells [21]. The negative relationship observed between mast cell counts and GMC:M ratio should be interpreted cautiously, as a decrease in relative count might not reflect an absolute decrease, but only a milder decrease compared to other cell populations. It is likely that a larger number of macrophages, and/or neutrophils are recruited compared to mast cells following an acute inflammatory event, even in the presence of a positive trend for all cell types. Absolute counts of BALF inflammatory cells as well as the knowledge of the volumes of saline-infused and recovered during the BAL procedure might help in the interpretation of relative counts and should always be considered in the future. Lastly, we acknowledge that most mastocytic MEA cases were from Turin. It is possible that the environment near Turin is characterized by pollutants or natural antigens driving BALF mastocytosis and GMC formation in the absence of disease in horses. This could have introduced a bias in our results. However, data from Montreal and Turin were coherent concerning the mast cell and GMC relationship observed. The only mastocytic MEA case in Montreal presented with increased GMC compared to neutrophilic MEA cases from the same site.

Alveolar macrophage counts (expressed in percentage) appeared to be negatively associated with age in our population, after appropriate adjustments, while no age-related effect was observed on GMC. Alveolar macrophage counts reduction with aging is coherent with immunosenescence, an age-associated deterioration in immunity characterized by a diminished reaction toward host pathogens [37]. It is described also in human beings [38]. Previous studies failed to detect any age-related decrease in alveolar macrophages in horses [37]. Further large-scale work is warranted to clarify this point. To the authors’ knowledge, whether GMC prevalence, phenotype or function is altered by age or by the aging environment is an unexplored research field. Age is a recognized risk factor for some GMC-mediated human diseases [39]. Age is also a well-recognized risk factor for SEA [40]. In addition, whether macrophage recruitment and its fusion (GMC formation) into the alveoli are driven by the same molecular pathways is largely undefined. Our data suggest that, in equine asthma, the two processes are regulated differently, and the assessment of these two cytological entities could provide complementary information from a clinical perspective.

## 5. Conclusions

In conclusion, our data support a possible role of GMC in equine asthma that deserves further attention. Their increased prevalence in SEA and their significant association with mast cells and, to a lesser extent, neutrophil relative cell counts in BALF, suggest they play a regulatory role in disease. This is in line with recent evidence showing alveolar macrophages, the precursors of GMC, as important orchestrators in equine asthma physiopathology. It must be remembered, however, that our results were obtained using relative ratios and this requires caution in data interpretation. Relative counts can indeed cause independently under or over-sized effects. 

Alveolar GMC might be increased in equine asthma in association with the increased burden of inhaled dust, due to altered mucociliary clearance, decreased respiratory flow, air trapping, or also other causes. A better knowledge of alveolar GMC prevalence, morphology, etiology, and function in asthma might disclose pathological mechanisms still undefined and deserves attention. Due to the high number of variables and biases that should be considered for this aim, large-scale work is warranted to gain meaningful insights and applicable results in the future.

## Figures and Tables

**Table 1:** Details of the horses studied.

	Turin Cohort	Montreal Cohort
	(N = 22)	(N = 20)
Controls		
N	0	10
Sex (M:F)	-	5:5
Age (years)	-	10.4 ± 3.6
Mild to moderate equine asthma (MEA)		
N	14	5
Sex (M:F)	8:6	3:2
Age (years)	7.4 ± 5.7	8.2 ± 1.6
Severe equine asthma (SEA)		
N	8	5
Sex (M:F)	6:2	3:2
Age (years)	15.6 ± 5.4	16.0 ± 4.5
Mastocytic asthma (M-MEA)		
N	7	1
Sex (M:F)	6:1	M
Age (years)	6.5 ± 7.5	8

MEA: mild to moderate equine asthma; M-MEA mastocytic mild to moderate equine asthma; SEA: severe equine asthma. M: male; F: female.

**Table 2:** Inflammatory signature of the horses studied.

	Paucigranulocytic	Neutrophilic ^1^	Mastocytic ^2^	Mixed
Controls	5	3	2	0
MEA	1	5	8	5
SEA	1	8	1	3

^1^ Cutoff for neutrophilia in controls and MEA = 5%, in SEA = 25%, respectively [1,2]. ^2^ Cutoff mastocytosis in all groups studied = 2%.

**Table 3:** Results of linear regression model used to investigate the relationship between GMC:M ratio and BALF cytological parameters, adjusting for age and for the presence and severity of clinical signs. Analyses were run on all asthmatic subjects (N = 32).

	β Coefficient	95% CI		*p* Value
BALF Neutrophil %	0.0002534	−0.0001471	0.0006538	0.20
BALF Mast cell %	−0.0037398	−0.0065036	−0.0009760	0.01
BALF Macrophage %	−0.0000191	−0.0004785	0.0004402	0.93
BALF Lymphocyte %	−0.0001442	−0.0005176	0.0002291	0.43
BALF Eosinophil %	−0.0003903	−0.0072857	0.0065051	0.91

BALF: bronchoalveolar lavage fluid. CI: confidence interval.

## Data Availability

Not applicable. All data produced in the study are presented in this article.
